# SLS-2 – the upgrade of the Swiss Light Source

**DOI:** 10.1107/S1600577518002722

**Published:** 2018-04-24

**Authors:** Andreas Streun, Terence Garvey, Lenny Rivkin, Volker Schlott, Thomas Schmidt, Philip Willmott, Albin Wrulich

**Affiliations:** a Paul Scherrer Institut, 5232 Villigen, Switzerland

**Keywords:** synchrotron radiation facility, electron storage ring, low-emittance lattice, undulator, imaging, molecular biology, X-ray spectroscopy

## Abstract

An upgrade of the Swiss Light Source (SLS) is planned for 2021–2024 and includes the exchange of the existing storage ring by a new one providing about 40–50 times lower emittance in user operation mode. This will extend the performance of SLS in particular in the fields of coherent imaging, full-field tomography, soft X-ray angle-resolved photoelectron spectroscopy and resonant inelastic X-ray scattering. A science case and a conceptual design for the machine have been established. As a summary of these reports, the novel lattice design, undulator developments and scientific highlights are presented.

## Abstract 2

The plans for an upgrade of the Swiss Light Source are presented, covering the storage ring lattice concept, the accelerator component designs, the insertion device developments and the science potential of the upgraded facility.

## 1. Introduction   

An upgrade of the Swiss Light Source (SLS), named SLS-2, is planned for the period 2021–2024 in order to increase brightness and the coherent fraction of the synchrotron light to a level competitive with the newest generation of light sources. The main component of the upgrade is the exchange of the existing electron storage ring for a new one providing 40 times lower emittance in user-operation mode.

A conceptual design report (Streun, 2017 ▸) as well as a scientific case (Willmott, 2016 ▸) have been established recently. In this paper we describe the concepts for the new storage-ring lattice and, for the most critical technical components, outline the further developments for insertion devices, and present promising experiments which will be enabled by the upgraded facility.

## 2. SLS history and status   

The SLS started user operation in June 2001 and has been operated in top-up mode since then. Today it is fully equipped with a set of 18 beamlines (11 based on undulators, three on super-bends and four on normal bends), covering areas as diverse as atomic and molecular science, catalysis/surface science, environmental and earth sciences, condensed-matter physics, life and medical sciences, materials science, and polymeric and soft-matter research. SLS delivers about 5000 h of user beam time per year at an availability of 97.6% (12-year average, 2005–2016), and excels in photon-beam stability (1 µm r.m.s. at the front-ends) and coupling control (lowest vertical emittance of 1 pm) (Aiba *et al.*, 2012 ▸).

The SLS storage ring is built from 12 triple-bend achromats (TBAs), connected by long (L), medium (M) and short (S) straights alternating as L–S–M–S, and thus has a threefold periodicity. The ring has a circumference of 288 m, and provides a horizontal emittance of 5 nm at 2.4 GeV. A chicane for generating 150 fs FWHM X-ray pulses by laser-beam slicing (at the FEMTO beamline) (Aiba *et al.*, 2013 ▸) increases the emittance to 5.6 nm, and further to ∼7 nm if operated. The insertion was installed in 2005 and has stopped operation recently since experiments migrated to SwissFEL, the Swiss Free-Electron Laser.

Between 2001 and the end of 2016, research output has produced 5373 peer-reviewed papers, including a uniquely high fraction of high-profile articles (1334 between an impact factor of 7.4 and below 25, and 298 with an impact-factor of 25 and higher).

In order to preserve the high scientific output of SLS, a significant reduction of the emittance is required, which promises sea-changes in many synchrotron-based scientific disciplines (Weckert, 2015 ▸). It comes at a highly opportune time, as it will subsume imminent maintenance and replacement of ageing components required at SLS anyway.

## 3. SLS-2 lattice concept   

The upgrade of the storage ring must address the issue of the comparatively small ring circumference, because, as a rule of thumb, emittance scales approximately inversely with the third power of the number of lattice cells (*i.e.* bending magnets with adjacent focusing elements) installed along the machine circumference. In the new generation of multi-bend-achromat (MBA) lattices, this number was increased through miniaturization of all components. Nonetheless, scaling existing designs like MAX IV (Leemann *et al.*, 2009 ▸), SIRIUS (Liu *et al.*, 2013 ▸) or ESRF-EBS (Farvaque *et al.*, 2013 ▸) to the circumference and energy of the SLS was insufficient to provide the desired emittance reduction; thus, a novel type of lattice was developed, which is based on the combination of longitudinal-gradient bends and reverse-bending magnets.

### 3.1. Radiation equilibrium   

The zero-current horizontal emittance in a storage ring is determined by the equilibrium between radiation damping and quantum excitation. The latter depends on the rate of photon emission, which is given by the strength of the magnetic field, and on the local dispersion function, because after emission of a photon an electron starts an oscillation around the closed orbit corresponding to its reduced energy. The damping of horizontal oscillations thus excited is given by the total radiated power, which depends on the magnetic field strength, and by the horizontal share in the overall damping, which is affected by transverse gradients in combined-function bending magnets. These relations are expressed by the radiation integrals 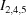 (Helm *et al.*, 1973 ▸): the horizontal emittance in a flat lattice (*i.e.* only horizontal bending magnets and no coupling) is given by the horizontal damping partitioning number,
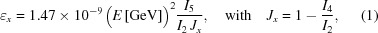
and *E* the beam energy.

### 3.2. Quantum excitation suppression   

The quantum excitation integral 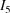 is given by
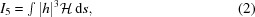
whereby *h* is the curvature (inverse radius) of the orbit in the magnetic field 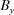,
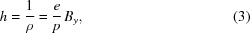
and 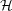 is the betatron amplitude of the dispersion function η,
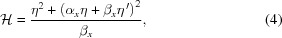
whereby β and α = 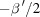 are the Courant–Snyder parameters (Courant & Snyder, 1958 ▸) and η and 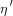 are the dispersion and its slope. 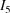 is minimized by using longitudinal-gradient bends (LGBs; bending magnets for which the field varies along the beam path) and by suppressing the dispersion at the LGB center, where the field is strongest. An optimal function 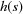 and optimal initial parameters 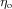 and 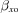 at the magnet center can be calculated to obtain the minimal 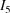 (or the minimal 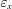), or a modified optimal function is obtained for given initial parameters (Streun & Wrulich, 2015 ▸). However, in a conventional periodic cell, quadrupoles are used to focus both 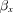 and η, which leads to an insufficient focusing of the dispersion function (or an over-focusing of 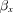), since the dispersion production of the bending magnet acts like a defocusing term, and thus the optimal dispersion 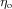 is out of reach. But the dispersion and β function can be decoupled by small reverse bends (RBs), *i.e.* bending magnets of opposite polarity and out of phase with the main bending magnet, which are realized by transverse displacement of the horizontal-focusing quadrupoles in order to obtain the desired dipolar down-feed (Streun, 2014 ▸).

### 3.3. Radiation damping enhancement   

The damping integrals 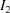 and 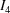 related to radiated power and damping partioning are given by
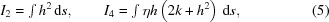
with 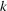 = 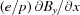, the quadrupole moment of a combined function bend. 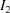 is enhanced in a lattice using LGBs and RBs, because the field variation in the LGB increases the radiated power compared with a homogeneous bending magnet, and the total absolute bending angle of the lattice is larger than 360°, due to the negative bending of the RBs. Furthermore, the horizontally focusing quadrupole component (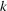 > 0) in the RBs (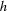 < 0) redistributes damping in favor of the horizontal dimension, *i.e.* 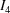 becomes negative and 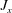 is increased.

Altogether, a lattice cell combining LGBs and RBs can provide up to a five times lower equilibrium emittance compared with a conventional cell (Streun *et al.*, 2017 ▸). This is an innovative and unique feature of the SLS-2 lattice.

## 4. SLS-2 lattice   

### 4.1. LGB–RB cell   

**Figure 1:**
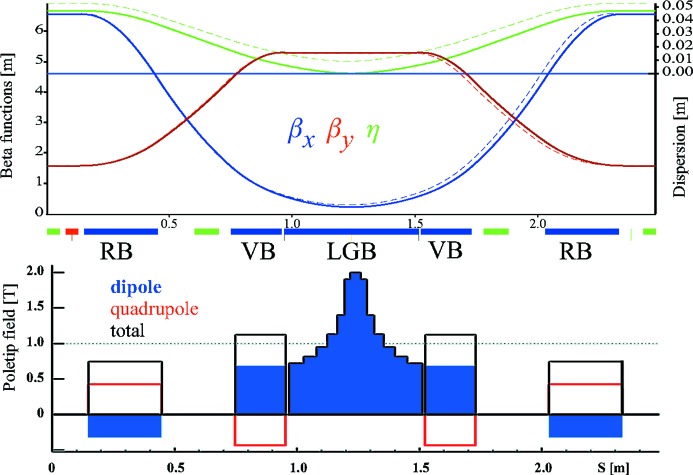
Optical functions and field components for the SLS-2 lattice cell containing a center LGB with adjacent vertically focusing bends (VBs), and two RBs. If the RBs were pure quadrupoles, the optical functions would follow the dashed lines in the upper plot and the emittance would be 4.5 time larger. The field components in the lower plot refer to the pole-tip field at 13 mm bore radius.

Fig. 1 ▸ shows the optical functions and the magnetic fields for the LGB–RB cell of SLS-2. The cell has a net bending angle of 5°, which is made up from the 6.4° deflection of the center LGB and the 2 × (−0.7°) deflection of the RBs. The LGB is realized as a compound magnet made from a pure dipole LGB of ∼4.4° sandwiched by two vertically focusing combined-function bends of 2 × (∼1°).

### 4.2. Lattice layout   

**Figure 2:**
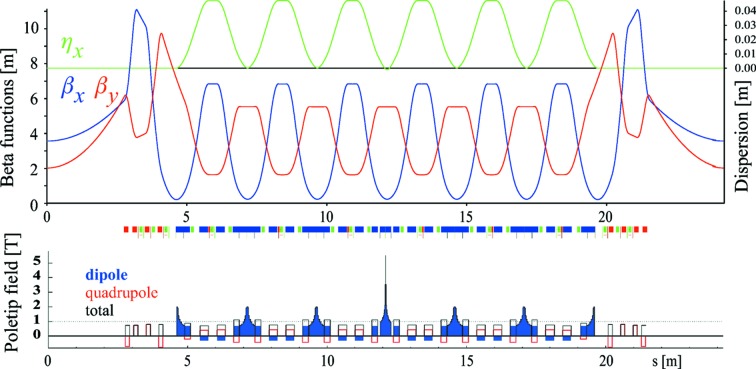
Optical functions and field components for one 7BA arc where the center LGB has been interchanged by a super-LGB of 5.5 T peak field. Bending magnets are in dark blue, quadrupoles in red and sextupoles in green.

Five full and two half LGB–RB cells form one of the 12 identical seven-bend achromat arcs (7BAs) as shown in Fig. 2 ▸, which replace the existing 12 TBA arcs of three different types.

Any of the LGBs can be exchanged by a LG-superbend of up to 6 T peak-field, in order to provide a brilliant source of X-rays in the 50–100 keV range.

All 12 straight sections are 5.5 m long. Eight full straights and three half straights are available for undulator installation, the others being required for injection elements and RF cavities. The higher lattice symmetry compared with SLS necessitates modifications of the lattice footprint, mainly affecting the regions of the present L-straights, while moderately affecting the others.

A comparison of the most important lattice parameters of SLS and SLS-2 is given in Table 1 ▸. For both rings, the beam energy is 2.4 GeV and the stored current is 400 mA. The increase of emittance and energy spread in SLS-2 due to intra-beam scattering is indicated in the table by arrows (→); this effect only becomes significant for low emittance, and is negligible for SLS. The SLS lattice is given in its present configuration, including three 2.9 T superbends and the chicane for laser-beam slicing (FEMTO), which will not be part of SLS-2. Three longitudinal gradient super-bends of 6 T peak-field are included in the SLS-2 lattice.

It is interesting to note that the momentum compaction factor α of the SLS-2 lattice becomes negative like in a proton synchrotron below transition energy, because the positive dependence of time-of-flight on momentum for high relativistic particles in the main bending magnets is overcompensated by RBs due to large dispersion and reversed polarity.

### 4.3. Acceptance   

Optimization of the dynamic acceptance is one of the most challenging problems for low-emittance lattices, because the strong horizontal focus in each bending magnet causes large chromaticity, and the small dispersion of short lattice cells results in very strong sextupoles for chromaticity correction.

For the dynamic acceptance optimization of the SLS-2 storage ring a robust design strategy was established, which is based on suppression of first- and second-order sextupole resonance-driving terms by phase cancellation: in the 7BA arc of SLS-2 this is achieved with cell tunes of 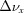 = 3/7 ≃ 0.429 and 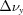 = 1/7 ≃ 0.143. The horizontal tune also enables the RBs to provide the most efficient emittance reduction by dispersion suppression in the LGB center. Nevertheless, cross-talk between the sextupoles causes large non-resonant second-order terms, which are amplitude-dependent tune shifts (ADTS) and second-order chromaticities. Suppression requires to back off the ideal cancellation pattern to some extent, and to fine-tune a total of seven sextupole and six octupole magnet families (Bengtsson & Streun, 2017 ▸).

The increase of the super-periodicity from three for the existing lattice with three different straight lengths to 12 in the SLS-2 lattice with only one type of straight was mainly motivated by dynamic acceptance optimization through elimination of potentially harmful resonances. Furthermore, identical arcs support standardization of components and ease of operation.

By these means, a relative momentum acceptance of about 5% was obtained which provides a Touschek-dominated beam lifetime of about 9 h (similar to SLS now), and a horizontal dynamic aperture of about ±6 mm at the point of injection, which enables off-axis injection, accumulation and top-up from the existing injector.

## 5. Technical systems   

### 5.1. Injection   

The booster synchrotron of the SLS was an innovative design at its time (Joho *et al.*, 2006 ▸). It anticipated features of modern storage rings, such as small-aperture magnets and many short lattice cells. Mounted to the inner wall of the storage-ring tunnel, the booster has a circumference of 270 m, which is 15/16 of the ring circumference, and delivers rather low emittances of 10 nm rad horizontal and 2 nm rad vertical at 2.4 GeV. These low emittances make it very suitable for off-axis injection into the smaller aperture of the SLS-2 storage ring. Therefore the SLS-injector will be reused for SLS-2 in its current form.

For top-up injection, the novel ‘anti-septum’ scheme was developed. It is based on an orbit bump formed by three dipole kickers, where a current sheet is placed inside the middle kicker to compensate the main field at the location where the injected beam passes (Gough & Aiba, 2017 ▸). Since the pulse of a kicker is weaker and shorter than in a septum magnet, the anti-septum can be as thin as 1 mm, thus reducing the distance between stored and injected beam and, with it, the aperture requirements.

### 5.2. Magnets   

Small apertures are a basic feature of low-emittance MBA lattices, because they enable an increase of magnet gradients and thus a reduction of magnet length and miniaturization of lattice cells. Many small magnets, in turn, result in small peak dispersion, such that sufficient momentum acceptance is still provided with small apertures. Due to miniaturization, the SLS-2 lattice contains 900 magnets in a 290.4 m circumference (of which 66 m are straights sections), which results in small inter-magnet distances and high fields and gradients.

The core component is a compound magnet containing the central LGB with about 2 T peak field, and two vertically focusing combined-function bending magnets (VBs) in a common yoke. The RBs are essentially radially shifted quadrupoles. Resistive coil and permanent-magnet designs are being evaluated in parallel for the RB and the LGB compound magnet, the final decision depending on costs and ongoing technological progress in the field. Modified versions of these magnets are used in the dispersion suppressor cells at the ends of the arcs, *i.e.* half-LGBs and RBs and VBs with modified gradients.

Any of the 60 LGBs may be exchanged by a LG-superbend; it is planned to start with three of these. The design is based on two pairs of coils, Nb_3_Sn racetrack-coils to create a narrow central field peak of 6 T, and NbTi Helmholtz coils to provide the required field integral (Calzolaio *et al.*, 2017 ▸).

Quadruplets of quadrupoles on both ends of the arcs are used for matching to the straight sections and provide margin to compensate any focusing from the insertion device.

A total of 288 sextupoles and 144 octupoles are employed for correction of chromaticity and optimization of acceptance. 120 horizontal and vertical orbit correctors are realized as additional coils in some of the sextupoles; 24 more in the straight sections are small dipoles and will be used to steer the photon beams from the undulators. Small quadrupoles for fine tuning and gradient corrections, and skew quadrupoles for coupling control, are realized as additional coils in some of the octupoles.

### 5.3. Beam stability   

Beam stability will be one of the key operational aspects for exploiting the improved brightness of SLS-2. For determination of the electron beam orbit, 144 button-type RF beam-position monitors (BPMs) will be installed in the storage ring. The beam positions will be measured with newly designed BPM electronics, which will provide <50 nm r.m.s. position noise for fast orbit feedback (FOFB) applications and <1 µm r.m.s. position resolution for turn-by-turn machine studies. By using all RF BPMs and all corrector magnets in the storage ring, a global orbit correction can be applied at a rate of 20 kHz, leading to a FOFB bandwidth of several hundred hertz when considering all latency contributions of the dedicated FOFB network. The SVD-based global FOFB algorithm will not only correct for orbit perturbations but will also provide the possibility of feed-forward corrections and user-defined adaptations of the reference orbit. The FOFB architecture will allow the implementation of additional feedback loops for, for example, betatron tune and beam size by integration of additional sensors such as photon-BPMs, coupling or beam size monitors and additional actuators such as normal and skew quadrupoles. Integration of photon-beam-based signals requires the development of reliable photon-BPMs, where a research and development program for SLS-2 is foreseen to make use of diamond membranes as quadrant detectors for hard X-rays and gas-based monitors for VUV and soft X-ray beamlines.

### 5.4. Radiofrequency   

SLS-2 will reuse the existing SLS RF system which consists of four 500 MHz copper cavities for a maximum sum voltage of 2.6 MV, and a passive superconducting third-harmonic (1.5 GHz) twin cavity for bunch lengthening and Landau damping. Since the momentum compaction of SLS-2 is negative and smaller than in SLS, the cavities will operate at the opposite slope of the RF wave for longitudinal focusing and at a reduced total voltage of 1.4 MV. For the main cavities, an absorber plug-in is proposed to damp some higher-order modes.

Due to its age, the RF system would benefit from extensive refurbishment, like replacement of klystrons by solid state amplifiers, and new low-level RF and cooling systems. Moreover, operation of the third-harmonic cavity in an active mode is under consideration to extend the flexibility in suppression of coupled bunch instabilities at lower current.

### 5.5. Vacuum system   

The vacuum chamber is based on a round beam pipe of 20 mm diameter. Sections with ante-chambers in the LGB regions, where radiation is emitted at high power, alternate with sections made from simple round pipes in the RB regions. The inner surface of the vacuum chamber is coated with non-evaporable getter (NEG) material, in order to suppress radiation-induced gas desorption from the chamber walls, and thus to establish a low average pressure of <10^−9^ mbar after only 100 Ah of beam dose. The NEG layer has a thickness of 1 µm in the ante-chambers but 500 nm only in the beam pipe, in order to reduce the resistive wall impedance.

The onset of turbulent bunch lengthening has been estimated, including impedance contributions from the vacuum chamber, BPMs, tapers and cavities. The resulting threshold current of 2 mA without and 3.5 mA with an ideal third-harmonic RF system for bunch lengthening are well above the desired beam current of about 1 mA per bunch (400 mA total current).

### 5.6. Mechanical engineering   

The magnets are mounted on girders, which are supported by concrete pedestals. The grouping of elements on girders and the mechanical design of the girders is guided by the requirements for stiffness and high eigenfrequencies and by the need for extensive preassembly in order to minimize the time required to mount the storage ring in the tunnel. Depending on the elements they carry, the girder supports will be equipped with manual adjustments or with motorized movers for remote, even beam-based, alignment. Irrespective of the final decision on the girder layout and adjustments, which will also be cost driven, the mechanical tolerance and stability requirements with regard to beam-dynamics performance can be met, and it is the subject of ongoing studies as to which extent remote-alignment capabilities may shorten the commissioning period and facilitate operation.

## 6. Insertion devices   

The SLS had been designed to cover a wide photon-energy spectrum from about 10 eV to 35 keV (excepting superbend radiation) in order to accommodate both hard and soft X-ray beamlines. The three types of straights with different lengths allow long-period electromagnetic undulators, APPLE II type undulators, and short-period in-vacuum undulators to be installed. The undulator gaps vary between 20 mm for the 8 m-long electromagnetic undulator UE212; 11 to 16 mm for the 4 m-long APPLE II undulators with 44 to 56 mm periods; and 4 mm for the 2 m-long in-vacuum undulators with 14 to 19 mm period length. All soft X-ray undulators provide variable polarization. The hard X-ray undulators operate at high harmonics. The standard undulators are operated at room temperature (RT) and have a 19 mm period, while a 14 mm period is realized with cryogenic undulators; hence the highest photon energies can be reached with high-brightness undulator radiation.

**Figure 3:**
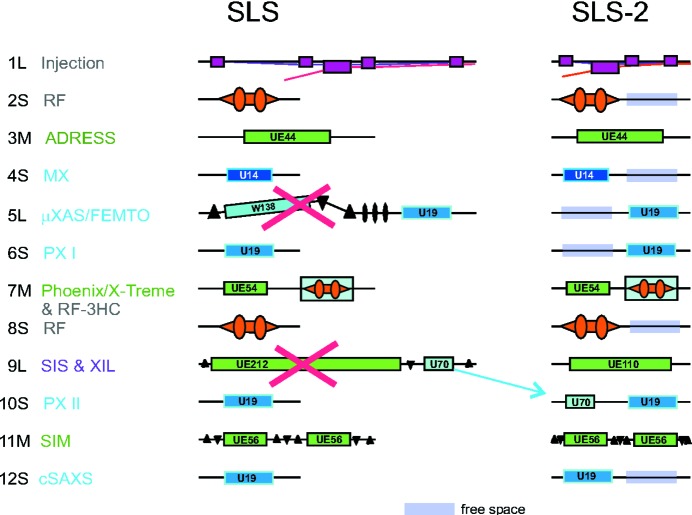
Occupancy of straight sections in SLS and in SLS-2.

The successful concept of the SLS will be adapted to SLS-2. The reconfiguration of the lattice with only one type of straight section with a maximum available space for undulators of about 4 m requires a new concept for the electromagnetic undulator. All other undulators could basically remain unchanged. However, in order to exploit the full potential of SLS-2, the undulators and the beamline optics have to be matched to the reduced emittance. In the process, new materials and conceptual designs have to be taken into account. Fig. 3 ▸ shows a possible layout of the straights in SLS-2, considering also options for canted undulators, or undulators and RF cavities sharing a straight.

In the following sections the options for undulator upgrades will be presented, grouped in soft X-ray, hard X-ray and the ‘tender’ X-ray regime, which is of growing interest.

### 6.1. Soft X-rays   

The SIS (Surface/Interface Spectroscopy; photon-energy range 10–800 eV) beamline is now served by a quasi-periodic electromagnetic undulator with variable periods of 212 mm and 424 mm. For SLS-2, a permanent-magnet undulator replacement is needed as, otherwise, within the available 4 m, there would be too few usable periods and hence insufficient flux. The reason for the long periods was to keep the *K*-value and the emitted power reasonably low. The quasi-periodicity shifts the harmonics to non-integer values so that they are effectively suppressed by the monochromator.

Harmonics and heat load are less of a problem in circular polarization mode, because only the fundamental is emitted on-axis. For linear polarization the ‘Figure-8’ undulator (Tanaka & Kitamura, 1995 ▸) was developed at SPring-8, which emits only a few harmonics on-axis and the higher harmonics into off-axis regions. A combined version is the ‘Helical-8’ undulator, proposed to provide all polarization modes with reduced heat load (Tanaka & Kitamura, 2011 ▸). Another option is the recently developed quasi-periodic ‘Knot-APPLE’ undulator (Sasaki *et al.*, 2014 ▸), which combines harmonic suppression with efficient reduction of on-axis power for all polarization modes. A first device has been realized (Ji *et al.*, 2015 ▸). However, quasi-periodic schemes in APPLE II structures can be optimized only for one polarization setting and are therefore not optimal.

Alternatively, twin elliptical undulators with opposite helicity and a proper phase control can be used to produce linear-polarized light of any polarization angle (Sasaki, 1997 ▸). Since the circular-polarized light contains no harmonics on-axis, the linear polarized light emitted on-axis also contains only the fundamental. This means that heat load at high *K*-values and harmonic contamination at low photon energies can be avoided. At higher photon energies, both undulators are operated phase-matched with the same helicity, or with opposite helicity, for fast helicity switching.

In crossed-undulator schemes, the degree of polarization has to be carefully considered. The degree of polarization reaches 80% but not over the full central cone. This results in a flux reduction of a factor of five at 12 eV. However, operation at high-*K*, low-photon energies and the small emittance should be best suited for the SIS beamline. In such a scenario an online polarization control is mandatory and is available with the single-shot polarimeter developed at DESY (Babenkov *et al.*, 2015 ▸).

The APPLE-X undulator design for the ATHOS beamline of SwissFEL can be reused, if the period is increased from 38 mm to about 90–100 mm (a modified version with a 90 mm period is already under development for the European XFEL). With a *K*-value of 10, the photon energy range from 12 to 600 eV can be covered at all linear- and circular-polarization modes. The highest photon energies of the beamline can be reached on the third harmonic.

The APPLE II type undulators UE56 and UE54 (56 and 54 mm period) presently in use at the SIM (Surface/Interface Microscopy) and X-treme (X-ray absorption spectroscopy at high magnetic fields and low temperature) beamlines can be used further at SLS-2 with only small modification: UE56 is a twin undulator with an electromagnetic chicane which has to be replaced with a much shorter permanent-magnet-based phase-matcher. UE54 is a single undulator, but used with variable polarization. Switching polarizations takes about 25 s. A hydraulic system of sub-micrometer precision with new electronic valves has been successfully tested. It would speed up polarization changes to a few seconds.

The soft X-ray beamline ADRESS (Advanced Resonance Spectroscopy) requires a high photon flux due to the required high resolution. The current undulator, UE44, is a 3.4 m-long fixed-gap APPLE II undulator with 75 periods of 44 mm, and an energy range from 180 eV to 2 keV. If an increase of the lowest photon energy to 400 eV (meaning that the carbon edge becomes inaccessible) is acceptable, operation of two units based on the above-mentioned SwissFEL/ATHOS design at a period of about 33 mm, and reducing the gap size, would increase the flux by a significant factor of 2.5 with respect to a continued use of UE44.

However, the ultimate solution for the ADRESS beamline would be the implementation of the echo-enabled harmonic generation (EEHG) scheme, proposed originally for use in FELs (Xiang & Stupakov, 2009 ▸), but also considered for use in storage rings (Evain *et al.*, 2010 ▸; Bakr *et al.*, 2011 ▸). The EEHG scheme causes coherent radiation by microbunching of the electrons at the resonant wavelength, and, although it is triggered by an external laser with a relatively low repetition rate in the kilohertz range, it could boost the brightness by up to four orders of magnitude.

### 6.2. Hard X-rays   

The SLS is equipped with four 2 m-long U19 in-vacuum undulators operated at RT. They provide photons energies from 5 to 20 keV using the third to 13th harmonics. The minimum gap is 4.5 mm, which cannot be lowered significantly in SLS-2 in order to prevent degradation of the elastic scattering beam lifetime and an increase of the corresponding Bremsstrahlung radiation levels.

The cryogenic undulator U14 (Calvi *et al.*, 2013 ▸), with a magnetic length of 1.7 m, can be operated down to a gap of 4 mm. Below this value radiation losses at the device increase significantly. SLS has only one vertical scraper in the injection straight, but two scrapers separated by an appropriate (*i.e.* approximately orthogonal) vertical betatron phase advance would be required to define the vertical acceptance. Without a second scraper, the undulator with the smallest gap [precisely, the smallest 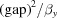 ratio] takes over this role. In order to enable safe operation at 4 mm gap, a second scraper will be mandatory for SLS-2.

**Figure 4:**
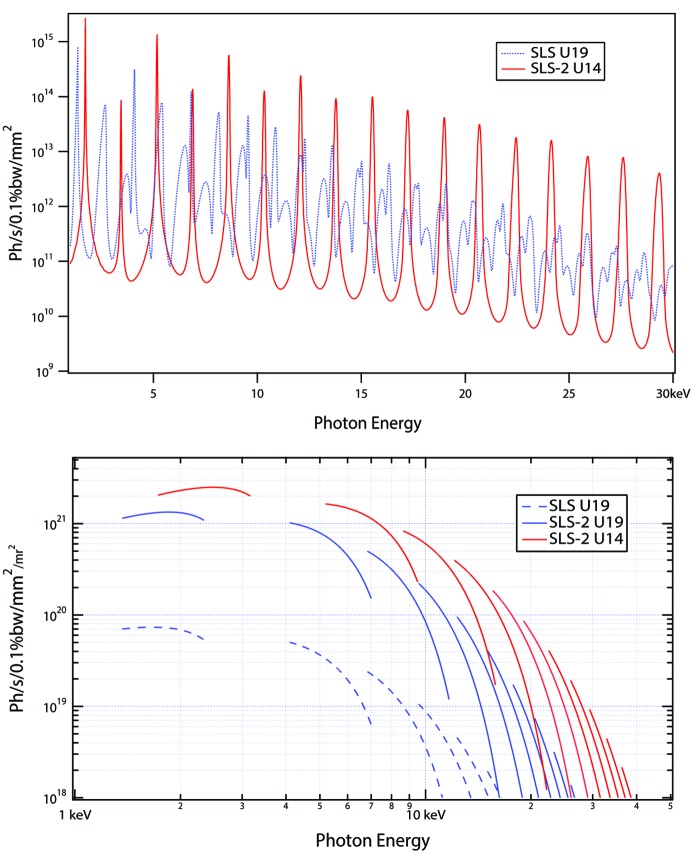

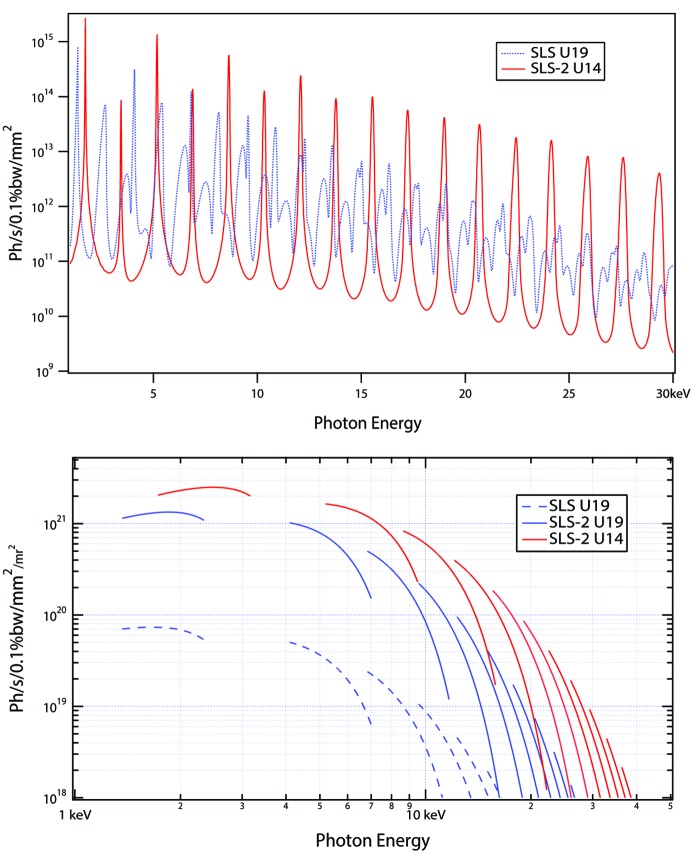
Flux-density (top) and brightness (bottom) for short-period in-vacuum undulators in SLS and SLS-2. Due to the low emittance of SLS-2, the harmonic spectrum will be much cleaner than at SLS.

Although the U19 undulator can be further used at SLS-2, new undulator technology would exploit the smaller emittance in order to boost the brightness or flux density, in particular for higher photon energies, as displayed in Fig. 4 ▸: the pole width could be reduced from 42 mm to 25 mm, following the reduction of the ‘beam stay clear’ aperture in SLS-2 from about ±32 mm to ±10 mm. Hence, stronger fields can be produced without increasing the overall forces on the support structure. Further, a new magnet material, PrFeB, has become available, which can be used in cryogenic undulators at liquid-nitrogen temperatures or even below. It does not show the phase transition at 135 K of the NdFeB material used in the existing cryogenic undulator CPMU14, which causes a precession of the magnetic vector with increasing opening angle at lower temperatures. Consequently, a simpler cooling concept could be established.

A possible upgrade scenario considers conversion of the existing U19 undulators to cryogenic undulators by only exchanging the magnet arrays and the vacuum components including the columns between the outer ‘I-beams’ (the pistons and bellows holding the magnet structures). In this manner, the excellent mechanics of the support structures and gap drivers can still be used, which will result in a cost-efficient upgrade. Only the electronic hardware requires refurbishment, which is the case for all undulators in SLS after 20 years of operation. The period lengths may be 14 mm or slightly below, depending on the required photon-energy range of the specific beamline. The energy gap between the first and third harmonic requires special attention (also see Fig. 4 ▸): for high photon energies shorter periods would be more effective, but a period length of about 14 mm is required to start on the third harmonic at a photon energy of 5 keV.

Superconducting undulators are also becoming a realistic alternative, whereas in the past the required tolerances on field and phase errors could not be achieved. Recent developments based on NbTi and Nb_3_Sn open possibilities for short-period undulators with high *K*-values, which are beyond the capabilities of permanent-magnet-based designs. Research and development on a high-field undulator is in progress for SwissFEL, which, if applied to SLS-2, would extend the energy spectrum into the 40–60 keV range. Due to the high brightness, this would enable an undulator-based microtomography beamline which would complement the superbend-based TOMCAT (TOmographic Microscopy and Coherent rAdiology experimenTs) beamline.

### 6.3. Tender X-rays   

One UE54 APPLE II type undulator sequentially serves the X-treme (see above) and Phoenix (Photons for the exploration of nature by imaging and XAS) beamlines. Phoenix covers the tender X-ray regime using the undulator up to the 30th harmonic. Obviously the beamline will be more efficient with an undulator of its own, which can be another UE54 or a dedicated undulator with a correspondingly shorter period.

### 6.4. Bending-magnet beamlines   

Presently, eight dipole beamlines are in operation at SLS, three are based on 2.9 T peak-field, normal-conducting, superbends, and five on regular dipoles of 1.4 T field.

The superbends will be exchanged by superconducting LGBs with a maximal peak field of up to 6 T, in order to extend the maximal photon energy from presently 45 keV to about 100 keV. The horizontal angle of acceptance for the beamlines is of the order of a few milliradians. In this range, the variation of the magnetic field of the super-LGB is still negligible.

The source points of most of the dipole beamlines are located in the dipole center; however, two of them have source points 4° upstream of the center. Since in the 7BA lattice the LGBs are shorter, these beamlines have to move to the adjacent upstream bend, which is separated by 5°.

## 7. Science   

This section has been adapted from the science part of the funding proposal submission to the Swiss National Science Foundation (SNF) for the SLS-2 upgrade in December 2017.

**Figure 5:**
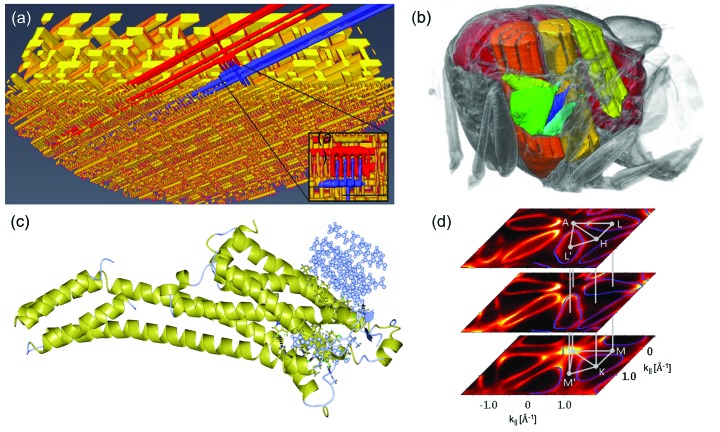
Examples of research performed at SLS. (*a*) Three-dimensional ptychographic tomographic imaging of an Intel processor with 15 nm resolution. [Reprinted by permission from Springer/Nature: Holler *et al.* (2017 ▸). *Nature (London)*, **543**, 402–406. Copyright (2017).] (*b*) Cutaway visualization of the thorax of a living blowfly (Walker *et al.*, 2014 ▸). (*c*) The high-resolution structure of the human adenosine A_2A_ GPCR (A_2A_R), solved using serial synchrotron crystallography (Weinert *et al.*, 2017 ▸). (*d*) The use of sufficiently high photon energies for which the increase of photoelectron escape depth translates, by the Heisenberg uncertainty principle, to a high intrinsic definition of the out-of-plane momentum, is important for the electronic structure resolution in all three dimensions (Strocov *et al.*, 2012 ▸).

The future scientific mission of photon science at the SLS-2 will have its foundations firmly based on already established fields of excellence at PSI. Among others, the SLS to date has produced world-leading research in activities as varied as ptychography, native-SAD, full-field tomography, soft-X-ray ARPES (angle-resolved photoelectron spectroscopy) and resonant inelastic X-ray scattering (Fig. 5 ▸). All of these techniques will profit considerably from the upgrade. Indeed, the enhanced brightness of SLS-2 will benefit almost all the X-ray techniques presently practiced at the SLS.

Here, some examples of recent studies at the SLS and how these will improve after the upgrade are presented as being particularly instructive and illustrative of the sea-changes promised by SLS-2. An exciting aspect of the upgrade is that the improvements extend beyond the increase in brightness alone, due to the enabling of other innovations, further down the technological chain and partly discussed in more detail in §6.

### 7.1. Imaging   

Imaging at the SLS includes several spectromicroscopies and scanning microspectroscopies, plus full-field tomography and ptychography.

Ptychography is a scanning variant of coherent X-ray diffractive imaging (CXDI), both of which depend intimately on the coherent part of the X-ray beam (which will increase in the hard X-ray regime by approximately a factor of 40 at SLS-2) to encode the necessary information to image objects. It bridges the resolution gap between crystal diffraction and electron microscopies on the angstrom and sub-angstrom scale, and nanoprobe methods such as scanning X-ray transmission microscopy (STXM), photoelectron emission microscopy (PEEM) and tomography on the tens of nanometers to micrometer scale.

Originally developed for electron microscopy, ptychography is now an emerging and increasingly popular and widely adaptable technique using X-rays, largely pioneered at the cSAXS beamline of the SLS. Its great strength is that it can image extended structures with nested architectures up to several micrometers and beyond, with a resolution of a few nanometers. A recent ptychography study from the cSAXS beamline involved reconstructing the internal three-dimensional architecture of an Intel chip with 15 nm resolution and over a 10 µm-diameter region (Holler *et al.*, 2017 ▸).

In addition to the increase in available coherent flux, 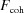, however, further improvements enabled by DLSR (diffraction-limited storage ring) technologies can be brought to bear, including (*a*) the installation of smaller-period undulators using high-remanence magnetic materials and ‘magnetic funnels’; (*b*) the use of the entire bandwidth of any particular undulator harmonic; and (*c*) improvements in the efficiency of X-ray optics. All told, the expected increase in available flux as a result of these enabling technologies is estimated to be in excess of 10000 compared with the current SLS. This has the potential to herald dynamics on the time-scale of minutes or even shorter.

This gain in flux can be invested in one or more of three channels: data-acquisition rates (frame rates and scanning velocities), spatial resolution (scattering vector, *Q*) and photon energy (which has an impact on the integrated sample dose, particularly important for biological and soft-matter samples), and will be tailored to individual experiments.

Full-field X-ray computed tomography (XCT) on the micrometer scale has burgeoned in the last decade and a half, not least in the fields of biological, palaeontological and industrial imaging. The TOMCAT beamline at the SLS has played a leading role in the development of XCT worldwide since the turn of the century.

The increased brightness from both undulators and high-field superbends at SLS-2 would allow XCT to be performed at much higher energies (with the associated penetration powers and lower integrated doses), and dramatically improve the dynamic capabilities on the micrometer scale (Walker *et al.*, 2014 ▸), which is expected to access the kilohertz range, potentially revolutionizing our understanding of countless life-science, industrial and advanced-manufacturing processes.

Phase-contrast XCT has proved to be especially powerful for low-dose experiments of weakly absorbing biological samples in the hard X-ray regime – the transverse coherence function of SLS-2 will be significantly more isotropic (*i.e.* the beam will be rounder). This will facilitate homogeneous edge-enhancement, and should thereby enable more sophisticated reconstruction algorithms that go beyond a linear approximation, which presently only makes use of the first-edge interference fringe. The larger coherent fraction will increase the phase contrast and thus also the information content that can be extracted per incident photon, plus it will allow one to move to higher photon energies for a given coherent fraction compared with the present. SLS-2 is therefore expected to strongly benefit phase-contrast imaging in all the variants offered at the SLS, including Talbot interferometry and Zernike techniques.

### 7.2. Molecular biology   

Third-generation synchrotrons have revolutionized modern macromolecular crystallography (MX). To date, well over 120000 atomic resolution biological structures have been deposited in the Protein Data Bank (http://www.pdb.org). However, one important class of protein, membrane proteins, including their subclass, G-protein-coupled receptors (GPCRs), which accounts for one-third of all proteins and two-thirds of medicinal drug targets, is extremely under-represented (1 to 2%). This is in a large part due to their hydrophobic nature, which makes crystallization difficult and normally limits crystal sizes to the micrometer scale. Such crystals require micrometer- or submicrometer beam focusing, which, until the advent of DLSRs, meant divergences in the horizontal plane that weakened the diffraction signal at high resolution. MX at SLS-2 will therefore concentrate on such systems, individually tailoring case-for-case the division of the improved emittance between divergence and focus size.

Due to the onset of radiation damage, individual microcrystals are insufficient for a complete data set, hence diffraction data from multiple crystals are merged (Smith *et al.*, 2012 ▸). Prompted by successes in serial femtosecond crystallography (SFX) at XFELs, there has been a thrust in the last five years towards similar approaches using synchrotron radiation in a technique coined synchrotron serial crystallography (SSX) (Diederichs & Wang, 2017 ▸). SSX can be carried out at room and cryogenic temperatures alike, requiring novel approaches to sample preparation, delivery, data collection and processing.

Despite the one to two orders of magnitude reduction in the highest tolerable dose compared with cryo-MX, RT-SSX offers several advantages, including sampling conformational landscapes, dispensing with cryoprotectants and, crucially, the possibility of investigating dynamic processes down to the microsecond time-scales (Panneels *et al.*, 2015 ▸). Moreover, and importantly with regard to the tight but quasi-parallel focusing attainable at SLS-2, it has been demonstrated that, in contrast to cryo-MX, there is a positive correlation between dose rate and maximum tolerable integrated dose in RT-SSX, which enables the measurement of more useful diffraction data by approximately a factor of six when increasing the dose rates from 0.5 to 5 MGy s^−1^ (Owen *et al.*, 2014 ▸).

With some notable but rare exceptions, the large majority of membrane proteins typically only produce weakly diffracting crystals with dimensions of the order of a few micrometers or still smaller. For these studies, the high brightness and small, parallel, beam of SLS-2 will be crucial in enhancing signal-to-noise ratios and reducing sample consumption. Since many membrane proteins are expected to be novel, experimental phasing is required to reveal their structures. Recent progress in native-SAD phasing has led to great advances in *de novo* phase determination (Liu & Hendrickson, 2015 ▸). SLS-2 will provide a timely opportunity to optimize existing MX beamlines for native-SAD experiments, which require X-rays down to 3 keV and sample environments with minimum background scattering and absorption (Wagner *et al.*, 2016 ▸).

The superior signal-to-noise ratio and the higher throughput of SSX that will be achieved with SLS-2 will be the key to unlocking the full potential of time-resolved studies. SSX, in combination with complementary XFEL studies, such as those possible at the sister facility SwissFEL, will thus continue to be the only reasonable techniques for protein dynamics on the millisecond to femtosecond timescale.

### 7.3. X-ray spectroscopies   

The majority of X-ray spectroscopies will profit from the smaller focal spots possible at SLS-2. At a typical undulator beamline, the horizontal source size will be approximately a factor of six smaller than at present. As the divergence is also approximately eight times smaller, the beam footprint in the *x*-direction will be correspondingly smaller, allowing any focusing optics to be positioned significantly further downstream, enabling more extreme demagnification and a more efficient use of circular diffraction-based optics such as Fresnel zone plates. This will allow soft-X-ray ARPES with nano­focusing, enabling *operando*- and domain-formation studies of emerging electronic materials (Strocov *et al.*, 2014 ▸).

Resonant X-ray inelastic scattering (RIXS) is a rapidly developing experimental method in which photons resonant with electronic transitions are inelastically scattered from matter (Ament *et al.*, 2011 ▸). The energy difference between the scattered and incident photon can range between meV and a few eV. This energy loss, plus the momentum transfer and change in polarization between the incoming and outgoing photon, can be directly associated with important processes related to emerging electronic and superconducting materials such as phonon-, magnon- or low-energy electron–hole-pair excitations.

The energy resolution of a RIXS experiment is determined by the spot size on the sample in the dispersive direction of an analysis grating spectrometer. The tighter focal spot-size of SLS-2 improves the resolution in principle by this amount. This will be further enhanced at SLS-2 by extending the beamline by over a factor of two to approximately 100 m, and increasing the demagnification of the incident spot size. In this manner, sub-10 meV resolution should be obtained, with the option of domain raster-scanning with submicrometer-sized beams on the sample and enabling the possibility of following, among other things, *operando* magnetic or ferroelectric domain switching.

Another interesting development is to disperse the incident radiation from the grating monochromator and image this onto the sample. Perpendicular to the dispersed incident beam, the focus remains tight, thereby preserving the resolution. By dispersing the scattered radiation with a second grating, the image formed on an area detector allows the parallel detection of both the incident- and inelastically scattered spectra in orthogonal directions (Strocov, 2010 ▸), enabling efficient, high-resolution, time-resolved experiments.

Hard X-ray scanning spectroscopies will also profit from the tight focuses possible at SLS-2. Moreover, line tomographies (which involve two transverse scanning coordinates and one rotational coordinate) will, like MX, take advantage of both tight and parallel beams (Corkhill *et al.*, 2017 ▸) down to the sub-micrometer scale. These ‘chemical imaging’ techniques exploit one or more of several signals (*e.g.* fluorescence, XANES, XRD) simultaneously, and can be applied to disciplines as broad as art history, cultural heritage, heterogeneous catalysis, battery technologies and environmental sciences.

## 8. Conclusion and outlook   

Most third-generation synchrotron facilities are either considering or actively undergoing an upgrade. It is imperative that the SLS does likewise. Novel machine elements pioneered at the PSI, in particular reverse bends and longitudinal gradient bends, enable an approximately factor of 50 improvement in horizontal emittance (zero current emittance), despite the small ring circumference. This will maintain the SLS as one of the best-performing synchrotron facilities worldwide for the foreseeable future.

The beamline upgrades will be in two phases. The first phase, in parallel with the machine upgrade, will encompass those beamlines most positively impacted by the increased brightness, probably including ptychography, full-field tomography, macromolecular crystallography and resonant inelastic X-ray scattering.

The SLS proposed upgrade Conceptual Design Report is being reviewed by the Swiss authorities to be included in the next ‘Swiss Research Infrastructures Roadmap’. Present planning foresees the realization of the project in the period 2021–2024.

A full technical design of all machine sub-systems will be completed over the next two years. Prototypes of critical components will be built and tested, and calls for tenders for large series components (magnets, vacuum chambers, girders *etc*.) will be prepared in 2020. Procurement, assembly and test of components are foreseen in the period 2021 to 2022. As there is a strong desire to minimize the down-time of the SLS, we plan to remove the present storage ring, replace it with the new ring and commission both the accelerator and beamlines in a period not exceeding 18 months (second quarter of 2023 to third quarter of 2024). Thus the SLS should be ready again for users before the end of 2024.

## Figures and Tables

**Table 1:** Main parameters for the SLS-2 upgrade lattice including three superbends in comparison with the existing SLS lattice The arrows (→) indicate the increase due to intra-beam scattering at a nominal current of 400 mA in 400 of 484 bunches for 10 pm of vertical emittance, and including a third-harmonic RF-system for bunch lengthening.

	SLS	SLS-2
Circumference (m)	288.0	290.4
Horizontal damping partition, *J*_*x*_	1.00	1.71
Momentum compaction, α	6.04 × 10^−4^	−1.33 × 10^−4^
Total *absolute* bending angle	374.7°	561.6°
Lattice tunes, ν_*x*_, ν_*y*_	20.4, 8.7	39.2, 15.3
Natural chromaticity, ξ_*x*_, ξ_*y*_	−67, −21	−95, −35
Radiated power (kW)	219.5	221.6
Emittance (pm)	5630	98 → 126
Energy spread (×10^−3^)	0.86	1.03 → 1.07
